# [Retracted] Epigenetic silencing of microRNA-199a-5p promotes the proliferation of non-small cell lung cancer cells by increasing AKAP1 expression

**DOI:** 10.3892/ol.2026.15784

**Published:** 2026-07-29

**Authors:** Nengli Yang, Yafeng Liang, Tianqi Zhu, Yanxiao Long, Zhe Chen, Xuezheng Zhang, Liuming Jiang

Oncol Lett 21: 434, 2021; DOI: 10.3892/ol.2021.12695

Following the publication of the above paper, it was drawn to the Editor's attention by a concerned reader that all of the data panels shown in Fig. 5D on p. 7, showing the results of EdU incorporation assay experiments, contained overlapping sections of data, such that the data were apparently derived from the same original source. Moreover, upon performing an independent analysis of the data in this paper in the Editorial Office, it came to light that western blot data featured in Fig. 5B (for the AKAP1 blots) had already appeared previously in an article written by different authors at different research institutes, which was published in the journal *Bioscience Reports*, and which itself has subsequently been retracted.

Owing to the fact that the contentious western blot data in the above article had already been published prior to its submission to *Oncology Letters*, the Editor has decided that this paper should be retracted from the Journal. Upon contacting the authors, they accepted the decision to retract the paper. The Editor apologizes to the readership for any inconvenience caused.

